# Microcirculatory disturbance in acute liver injury is triggered by IFNγ-CD40 axis

**DOI:** 10.1186/s12950-024-00387-w

**Published:** 2024-06-21

**Authors:** Miho Kurokawa, Takeshi Goya, Motoyuki Kohjima, Masatake Tanaka, Sadahiro Iwabuchi, Shigeyuki Shichino, Satoshi Ueha, Tomonobu Hioki, Tomomi Aoyagi, Motoi Takahashi, Koji Imoto, Shigeki Tashiro, Hideo Suzuki, Masaki Kato, Shinichi Hashimoto, Hideo Matsuda, Kouji Matsushima, Yoshihiro Ogawa

**Affiliations:** 1 Department of Medicine and Bioregulatory Science, Graduate School of Medical Sciences, Kyushu University, 3-1-1 Maidashi, Higashi-ku, Fukuoka, 812-8582 Japan; 2 Department of Molecular Pathophysiology, Institute of Advanced Medicine, Wakayama Medical University, 811-1 Kimiidera, Wakayama-shi, 641-8509 Japan; 3 Division of Molecular Regulation of Inflammatory and Immune Diseases, Research Institute for Biomedical Sciences, Tokyo University of Science, 2641 Yamazaki, Noda, 278-8510 Japan; 4 Department of Bioinformatic Engineering, Graduate School of Information Science and Technology, Osaka University, 1-5 Yamadaoka, Suita-shi, 565-0871 Japan; 5 Graduate School of Nutritional Sciences, Nakamura Gakuen University, 5-7-1 Befu, Jounan-ku, Fukuoka, 814-0198 Japan; 6 Department of Gastroenterology, NHO Kyushu Medical Center, 1-8-1 Jigyohama, Chuo-ku, Fukuoka, 810-8563 Japan; 7 Department of Gastroenterology and Hepatology, NHO Fukuokahigashi Medical Center, 1-1-1 Chidori, Koga, 811-3195 Japan

**Keywords:** Sinusoidal hypercoagulation, Tumor necrosis factor-alpha, Innate lymphoid cell, Liver sinusoidal endothelial cell, Resident macrophage

## Abstract

**Background:**

Acute liver failure (ALF) is a life-threatening disorder that progresses from self-limiting acute liver injury (ALI). Microcirculatory disturbance characterized by sinusoidal hypercoagulation and subsequent massive hypoxic hepatocyte damage have been proposed to be the mechanism by which ALI deteriorates to ALF; however, the precise molecular pathway of the sinusoidal hypercoagulation remains unknown. Here, we analyzed ALI patients and mice models to uncover the pathogenesis of ALI with microcirculatory disturbance.

**Methods:**

We conducted a single-center retrospective study for ALI and blood samples and liver tissues were analyzed to evaluate the microcirculatory disturbance in ALI patients (*n* = 120). Single-cell RNA sequencing analysis (scRNA-seq) was applied to the liver from the concanavalin A (Con A)‑induced mouse model of ALI. Interferon-gamma (IFNγ) and tumor necrosis factor-alpha knockout mice, and primary human liver sinusoidal endothelial cells (LSECs) were used to assess the mechanism of microcirculatory disturbance.

**Results:**

The serum IFNγ concentrations were significantly higher in ALI patients with microcirculatory disturbance than in patients without microcirculatory disturbance, and the IFNγ was upregulated in the Con A mouse model which presented microcirculatory disturbance. Hepatic IFNγ expression was increased as early as 1 hour after Con A treatment prior to sinusoidal hypercoagulation and hypoxic liver damage. scRNA-seq revealed that IFNγ was upregulated in innate lymphoid cells and stimulated hepatic vascular endothelial cells at the early stage of liver injury. In IFNγ knockout mice treated with Con A, the sinusoidal hypercoagulation and liver damage were remarkably attenuated, concomitant with the complete inhibition of CD40 and tissue factor (TF) upregulation in vascular endothelial cells. By ligand-receptor analysis, CD40-CD40 ligand interaction was identified in vascular endothelial cells. In human LSECs, IFNγ upregulated CD40 expression and TF was further induced by increased CD40-CD40 ligand interaction. Consistent with these findings, hepatic CD40 expression was significantly elevated in human ALI patients with microcirculatory disturbance.

**Conclusion:**

We identified the critical role of the IFNγ-CD40 axis as the molecular mechanism of microcirculatory disturbance in ALI. This finding may provide novel insights into the pathogenesis of ALI and potentially contribute to the emergence of new therapeutic strategies for ALI patients.

**Supplementary Information:**

The online version contains supplementary material available at 10.1186/s12950-024-00387-w.

## Background

Acute liver failure (ALF) is a life-threatening systemic disorder characterized by severe coagulopathy and encephalopathy [1, 2]. Although patients with ALF undergo multi-disciplinary therapies, including plasma exchange and blood purification, liver transplantation was only proved to improve the patients' prognosis [3, 4], partly due to the incomplete understanding of the pathogenesis of ALF. ALF is considered to progress from self-limiting acute liver injury (ALI), and massive hepatic necrosis is the characteristic histological feature of ALF. A substantial number of studies, including ours, have reported that microcirculatory disturbance characterized by sinusoidal hypercoagulation and consequent parenchymal hypoxic damage progressed in a significant proportion of patients with ALI [5–10]. Hypoxic condition is well known to upregulate lactate dehydrogenase (LDH) expression in diverse cell types [11, 12], and the serum alanine aminotransferase (ALT)/LDH ratio was reported as a sensitive marker of hypoxic hepatitis [13, 14]. We applied this ALT/LDH ratio to patients with ALI and confirmed histologically that sinusoidal fibrin deposition and the expressions of tissue factor (TF) and hypoxia-related proteins significantly increased in the sinusoidal microcirculatory disturbance (SMD) group (ALT/LDH ratio ≤ 1.5) than in non-sinusoidal microcirculatory disturbance (NSMD) group (ALT/LDH ratio > 1.5) [10]. It is also confirmed that the Concanavalin A (Con A) induced ALI model presented microcirculatory disturbance [15–17], and tumor necrosis factor-alpha (TNFα)/galactosamine (GalN) ALI model mimicked ALI without microcirculatory disturbance [10]. Recently, it was elucidated that cytokines, especially interferon-gamma (IFNγ) and TNFα, released from excessively activated immune cells play crucial roles in sinusoidal hypercoagulation in Con A model [17, 18]; however, exact cellular sources and targets of cytokines and the precise molecular mechanism of sinusoidal hypercoagulation remain unknown.

In this study, we analyzed ALI patients and the Con A mice model to investigate the consequence and mechanism of IFNγ for microcirculatory disturbance. Our findings may provide novel insights into the pathogenesis of ALI and potentially contribute to the emergence of new therapeutic strategies for ALI patients.

## Results

### Consequence of IFNγ for microcirculatory disturbance in acute liver injury

**Fig. 1:**
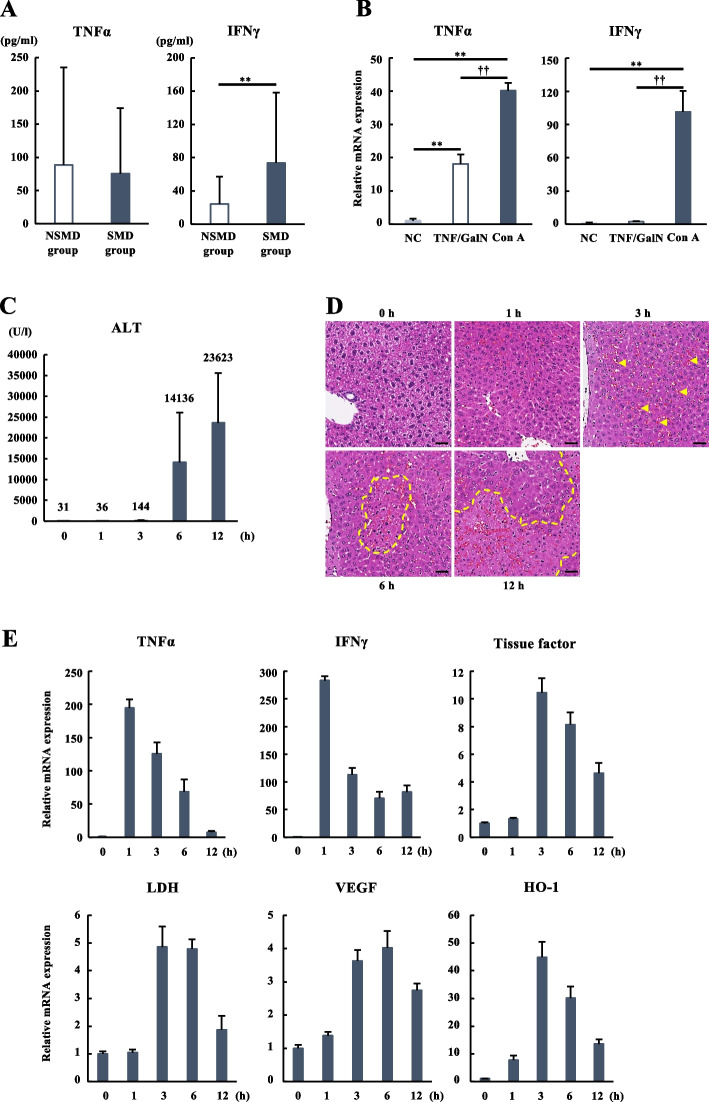
Consequence of IFNγ for microcirculatory disturbance in acute liver injury. **A** Serum TNFα and IFNγ levels in the SMD and NSMD groups. The data were expressed as mean and SD. **P < 0.01 when compared to the NSMD group. **B** RT-qPCR analysis of TNFα and IFNγ gene expression in Con A (15mg/kg) and TNF/GalN (700 mg/kg GalN +15 µg/kg TNFα) livers. The gene expression levels were normalized to those in the untreated mice as NC. The data were presented as mean and SE (*n* = 3–5 in each group). **P < 0.01 vs. NC groups and ^††^*P* < 0.01 vs. TNF/GalN group. **C** Serum ALT levels after 0, 1, 3, 6, and 12 hours of Con A administration. The data were expressed as mean and SD (*n* = 6–13 in each group). **D** Hematoxylin and eosin staining of the livers after 0, 1, 3, 6, and 12 hours of Con A administration. The dashed area indicates necrotic features and arrowheads indicate hemostasis. Scale bars = 50 μm. **E** RT-qPCR analysis of the genes associated with the inflammatory cytokines, coagulation, and hypoxia in Con A livers (25mg/kg Con A). The gene expression levels were normalized to those at 0 hour. The data were expressed as mean and SE (*n* = 3–8 in each group). ALT, alanine aminotransferase; Con A, concanavalin A; GalN, galactosamine; HO-1, heme oxygenase 1. IFNγ, interferon-gamma; LDH, lactate dehydrogenase; NC, normal control; NSMD, non-sinusoidal microcirculatory disturbance; RT-qPCR, quantitative reverse transcription polymerase chain reactions; SMD, sinusoidal microcirculatory disturbance; TNFα, tumor necrosis factor-alpha; VEGF, vascular endothelial growth factor

We have previously demonstrated that intrahepatic microcirculatory disturbance due to sinusoidal hypercoagulation caused parenchymal hypoxia in patients with ALI, and the sinusoidal microcirculatory disturbance could be identified using the ALT/LDH ratio [10]. Because TNFα and IFNγ were reported to be crucial for Con A-induced ALI in mice [18], we measured these cytokines in the serum of 120 patients with ALI categorized by the presence of the microcirculatory disturbance. Thirty-eight patients were classified as SMD group (ALT/LDH ratio ≤ 1.5) and 82 as NSMD group (ALT/LDH ratio > 1.5) (Table 1A). Given that the serum TNFα and IFNγ concentrations in healthy volunteers were reported to be less than 1.0 pg/ml [19], those were markedly elevated in both SMD and NSMD groups (Fig. 1A). Serum TNFα levels had no significant difference between SMD and NSMD groups (NSMD vs. SMD: 88.5 ± 146.9 pg/ml vs. 75.4 ± 98.8 pg/ml), while serum IFNγ level was higher in the SMD group than in the NSMD group (NSMD vs. SMD: 24.7 ± 39.7 pg/ml vs. 68.4 ± 83.4 pg/ml) (Fig. 1A). No correlation was found between serum IFNγ and serum ALT levels, implying that elevated serum IFNγ levels did not reflect the severity of the liver injury. Then, we compared the hepatic gene expressions of TNFα and IFNγ between two distinct mouse models of ALI, a Con A model with microcirculatory disturbance and a TNFα/GalN model without microcirculatory disturbance. The TNFα expression was significantly elevated in both Con A and TNF/GalN models relative to normal controls (NC), but more notably in the Con A model (Fig. 1B). The IFNγ expression was markedly increased in the Con A model, while IFNγ expression in the TNF/GalN model was almost unchanged, suggesting the involvement of IFNγ in the sinusoidal microcirculatory disturbance. Furthermore, we evaluated the temporal changes of hepatic gene expressions of cytokines, coagulation factors, and hypoxia-related proteins by the quantitative reverse transcription polymerase chain reactions (RT-qPCRs) and analyzed their correlation with serum liver enzyme and liver histology in the Con A model. The accession of serum ALT level was detected 3 hours after the Con A injection, and the ALT was steadily increased in a time-dependent manner (Fig. 1C). Histological analysis showed that a mild degree of sinusoidal congestion appeared at 3 hours after Con A administration, necrotic areas developed at 6 hours and expanded afterwards, and the hemorrhagic necrosis was extensive at 12 hours (Fig. 1D). Surprisingly, the hepatic IFNγ and TNFα expression levels were significantly increased as early as 1 hour after Con A administration when no liver damage was detected and then rapidly decreased at 6 or 12 hours when the massive liver injury was expanded (Fig. 1E). The gene expression levels of TF, a primary initiator of the coagulation cascades, and hypoxia-related genes including LDH, vascular endothelial growth factor (VEGF), and heme oxygenase 1(HO-1) were elevated 3–6 hours after Con A administration, reflecting the microcirculatory disturbance (Fig. 1E). The elevation of hepatic IFNγ and TNFα expressions preceded the sinusoidal hypercoagulation, hypoxic changes, and serological and histological liver injury in the Con A model, suggesting that these cytokines triggered the microcirculatory disturbance in ALI.

**Table 1:** Characteristics of patients with ALI at the time of admission

All population	NSMD group (*n* = 82)	SMD group (*n* = 38)	*p*-value
Age	45 (16–82)	46 (18–81)	0.95
Sex (M/F)	44/38	22/16	0.66
Etiology			
HAV, HBV, AIH, DILI, UK, and others	11, 30, 11, 4, 16, 10	8, 7, 0, 2, 16, 5	0.017
MELD score	16 (12–20)	18 (11–29)	0.083
Surviving/death and LT	70/11	37/1	0.064
ALI / ALF without coma / ALF with coma	31/43/8	5/29/4	0.021
Plt (/μl)	15.9 (11.3–20.8)	11.8 (9.4–14.8)	0.0003
PT (%)	46 (33–61)	35.5 (22–44)	0.0002
PT-INR	1.61 (1.31–2.16)	1.95 (1.65–2.82)	0.005
FDP (μg/ml)	7.9 (3.3–15.4)	27.6 (15.4–53.2)	<0.0001
AST (IU/l)	1503 (750–3259)	6964 (4563–12649)	<0.0001
ALT (IU/l)	2563 (1047–4089)	4804 (3208–6784)	<0.0001
LDH (IU/l)	571 (409–1318)	4893 (3458–10049)	<0.0001
Alb (mg/dl)	3.5 (3.2–3.9)	3.7 (3.5–3.9)	0.059
TB (mg/dl)	5.6 (3.6–12.5)	2.9 (1.7–4.3)	<0.0001
Cre (mg/dl)	0.67 (0.56–0.85)	0.9 (0.64–1.36)	0.039
NH_3_ (mg/dl)	65 (50–95)	67 (53–82)	0.82
Ferritin (ng/ml)	3808 (1178–7529)	25280 (6186–59215)	<0.0001

Data were expressed as median and interquartile ranges

*AIH* Autoimmune hepatitis, *Alb* Albumin, *ALF* Acute liver failure, *ALI* Acute liver injury, *ALT* Alanine aminotransferase, *AST* Aspartate aminotransferase, *Cre* Creatinine, *DILI* Drug-induced liver injury, *FDP* Fibrin degradation products, *LDH* Lactate dehydrogenase, *LT* Liver transplantation, *MELD* Model for end-stage liver disease, *NH*_*3*_ Ammonia, *NSMD* Non-sinusoidal microcirculatory disturbance, *Plt* platelet, *PT%* Prothrombin activity percentage, *PT-INR* Prothrombin time-international normalized ratio, *SMD* Sinusoidal microcirculatory disturbance, *TB* Total bilirubin, *UK* Unknown etiology

### Origins and targets of IFNγ and TNFα identified by single-cell RNA-seq analysis

**Fig. 2:**
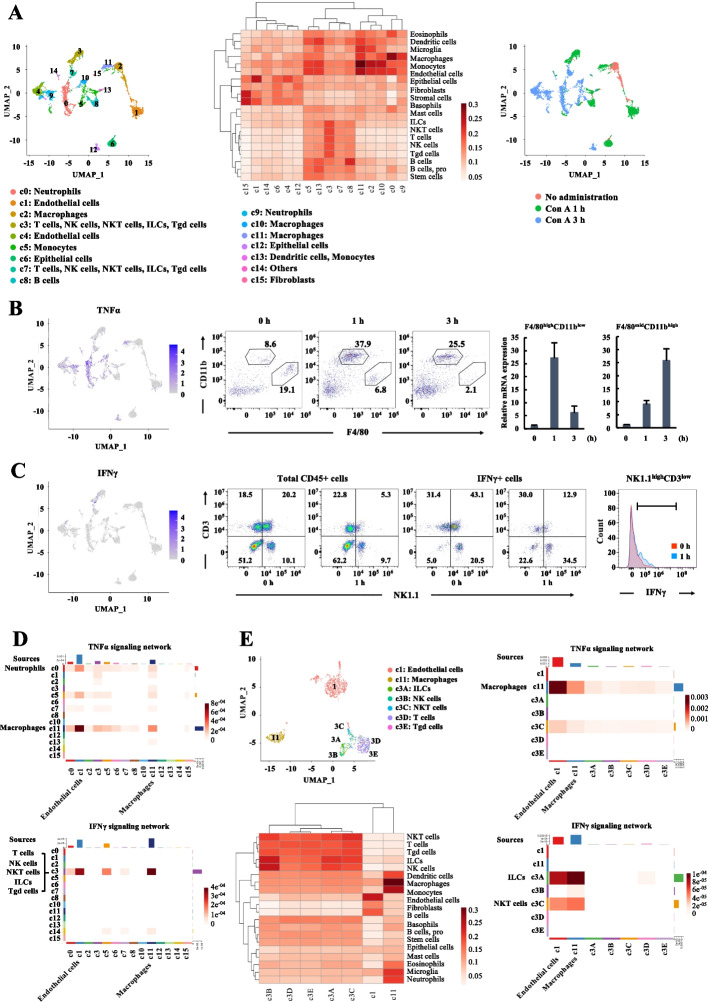
Origins and targets of IFNγ and TNFα identified by single-cell RNA-seq analysis. **A** The UMAP visualization of 5,478 cells from three livers, the clusters and sample origins were distinguished by colors. Heatmaps showed cell distribution between labels and clusters, and color scale expressed as log10-number of cells. **B** TNFα expression patterns were displayed on the UMAP plot. The legend represents the relative expression of each marker, from the lowest (gray dots) to the highest expression (blue dots). Flow cytometry plots showed the percentage of macrophages among CD45-positive cells extracted from Con A liver tissues. The TNFα gene expression was investigated by RT-qPCR in two cell populations of macrophages. The gene expression levels were normalized to those at 0 hour. The data were presented as mean and SE (*n* = 3 in each group). **C** IFNγ expression patterns were displayed on the UMAP plot. CD45-positive cells were divided into four groups based on CD3 and NK1.1 expression. Left flow cytometry plots showed the percentage of four groups at 0 and 1 hour after Con A administration. The percentage of IFNγ-expressing cells in the four groups was demonstrated in Right flow cytometry plots. The histograms depict 0 hours (dashed red line) and 1 hour (solid blue line) in NK1.1^high^CD3^low^ population. **D** The heatmap shows the relative strength of the TNFα and IFNγ signaling pathway networks for each cluster. **E** The distribution of vascular endothelium, macrophages, and T lymphocytes, with c3 re-clustered, is presented on the UMAP plot. The heatmap shows the relative strength of the TNFα and IFNγ pathway networks for each cluster. Con A, concanavalin A; IFNγ, interferon-gamma; ILC, innate lymphoid cell; NK cell, natural killer cell; NKT cell, natural killer T cell; RT-qPCR, quantitative reverse transcription polymerase chain reactions; Tgd cell, gamma delta T cell; TNFα, tumor necrosis factor-alpha; UMAP, uniform manifold approximation and projection

To identify the origins and targets of IFNγ and TNFα, the single-cell RNA sequencing analysis (scRNA-seq) was conducted on hepatic nonparenchymal cells (NPCs) isolated from mice in a healthy group, 1 hour (Con A 1h) and 3 hours (Con A 3h) after Con A administration. After sequencing, aggregation of the samples, quality control, and the exclusion of cells resembling doublets, a total of 5,478 single-cell transcriptomes from healthy controls, Con A 1h, and Con A 3h were collected and analyzed. The single cells were clustered by Seurat analysis and visualized by a uniform manifold approximation and projection (UMAP). We identified 16 distinct clusters, including vascular endothelial cells (cluster 1,4), macrophages (cluster 2, 10, 11), T cells/natural killer cells (NK cells)/ natural killer T cells (NKT cells)/ innate lymphoid cells (ILCs) (cluster 3, 7), B-cells (cluster 8), neutrophils (cluster 0, 9), monocytes (cluster 5), dendritic cells (DCs) (cluster 13), epithelial cells (cluster 6, 12), fibroblasts (cluster 15), and others (cluster 14), which were annotated by SingleR analysis (Fig. 2A, Fig. S1). Except for cluster 2, the majority of the immune cells infiltrated following Con A administration. The TNFα expression was detected in a wide range of clusters, including cluster 0, 9, 10, and 11, which correspond to macrophages in the Con A 1h group and neutrophils and macrophages in the Con A 3h group (Fig. 2B). To explore more specific cellular sources of TNFα, we isolated and analyzed NPCs from the livers of Con A 1h and Con A 3h by flow cytometry and cell sorting. The resident macrophages (indicated by F4/80^high^CD11b^low^) were reduced early after Con A administration, while recruited macrophages (indicated by F4/80^mid^CD11b^high^) increased over time (Fig. 2B). RT-qPCR analysis using RNA extracted from each cell population demonstrated that upregulated TNFα expression was evident 1 hour after Con A treatment in the F4/80^high^CD11b^low^ resident macrophage. In contrast, TNFα expression increased 3 hours after Con A administration in the F4/80^mid^CD11b^high^ cells, indicating that the source of TNFα shifted from resident macrophages to recruited macrophages (Fig. 2B). The IFNγ expressing cells were mainly located in cluster 3, containing T cells/NK cells/NKT cells/ILCs in Con A 1h (Fig. 2C). Flow cytometric analysis showed that the proportion of the NKT cells (indicated by NK1.1^high^CD3^high^) was drastically decreased at 1h after Con A treatment. In contrast, the composition rate of the T cells (indicated by NK1.1^low^CD3^high^) and the NK cells/ILCs (indicated by NK1.1^high^CD3^low^) were almost unchanged (Fig. 2C). To determine the cellular source of IFNγ, we isolated the NKT cells, the T cells, and the NK cells/ILCs by cell sorting and analyzed IFNγ gene expression by RT-qPCR; however, the upregulation of IFNγ was not prominent in every cell population (Fig. S2). In our experimental protocol, the handling time from the extraction of mouse livers to the cell isolation by FACS was approximately 8 hours; we presumed that the instability of the IFNγ transcript might affect the results. We assessed the cellular source of IFNγ using the PrimeFlow assay, an in situ hybridization assay which enables the simultaneous detection of intracellular RNA and cell surface protein expressions by flow cytometry [20], to minimize the degradation of the IFNγ transcript. The proportion of IFNγ-expressing NK cells/ILCs (NK1.1^high^CD3^low^) markedly increased 1 hour after Con A treatment (20.5% to 34.5%), and the proportion of IFNγ-expressing NKT cells (NK1.1^high^CD3^high^) decreased at 1 hour after Con A administration (43.1% to 12.9%), which might be attributed to the decreased proportion of total NKT cells irrespective of IFNγ expression, indicating NK1.1^high^CD3^low^ cell population might be the source of IFNγ (Fig. 2C). To determine the cellular origins and targets of cytokine signaling, scRNA-seq ligand-receptor analysis was conducted using the data at 1 hour after Con A administration. We identified that TNFα and IFNγ signaling originated mainly in cluster 11 (macrophages) and cluster 3 (T cells/NK cells/NKT cells/ILCs), respectively. More importantly, the primary target cells of both TNFα and IFNγ signals were cluster 1 (vascular endothelial cells) and cluster 11 (macrophages) (Fig. 2D). To investigate more detailed cell-cell interactions of clusters 1, 3, and 11, we extracted and re-clustered these cells and found that the cluster 3 which is the cellular origin of IFNγ, comprised distinct five lymphocyte populations (Fig. 2E, Fig. S3). SingleR analysis suggested that cluster 3A, 3B, 3C, 3D, and 3E were related to ILCs, NK cells, NKT cells, T-cells, and gamma delta T-cells (Tgd cells), respectively. Cluster 11 (macrophages) predominantly expressed TNFα and interacted with cluster 1 (vascular endothelial cells) and 11 (macrophages). In comparison, cluster 3A (ILCs) and cluster 3C (NKT cells) highly expressed IFNγ and targeted cluster 1 (vascular endothelial cells) and 11 (macrophages) (Fig. 2E, Fig. S4). Because the PrimeFlow assay showed that IFNγ-expressing NK1.1^high^CD3^low^ cell population (NK cells or ILCs) increased and IFNγ-positive NK1.1^high^CD3^high^ cell population (NKT cells) decreased at 1 hour after Con A administration, it is highly conceivable that the primary source of IFNγ was ILCs, rather than NKT cells. Furthermore, it was noteworthy that vascular endothelial cells were the targets of both TNFα and IFNγ signaling.

### Requirement of IFNγ in microcirculatory disturbance

**Fig. 3:**
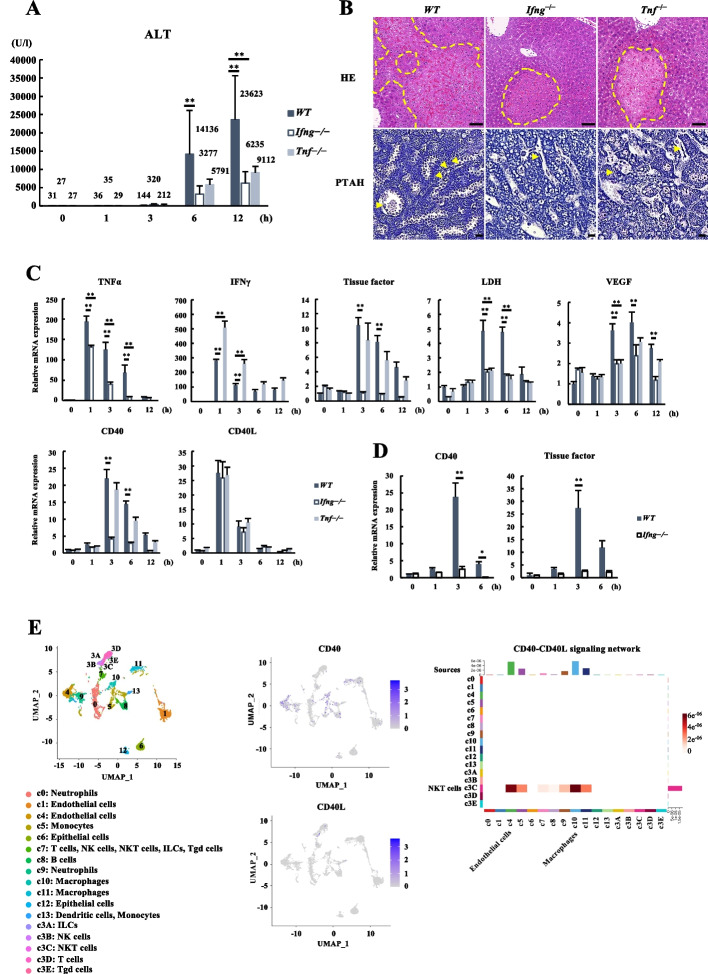
Requirement of IFNγ in microcirculatory disturbance. **A** Serum ALT levels 0, 1, 3, 6, and 12 hours after Con A administration in the *WT*, *Tnf*^–/–^, and *Ifng*^–/–^ mice. The results were expressed as mean and SD (*n* = 6–13 in each group). **P < 0.01 when compared to the *WT* mice. **B** Hematoxylin and eosin staining and PTAH staining of the liver for the *WT*, *Tnf*^–/–^, and *Ifng*^–/–^ mice. The dashed area indicates necrotic features and arrowheads indicate fibrin depositions in sinusoids. Scale bars = 50 μm. **C** RT-qPCR analysis of the genes of the inflammatory cytokines, coagulation, and hypoxia in the liver of *WT*, *Tnf*^–/–^, and *Ifng*^–/–^ mice treated with Con A. The gene expression levels were normalized to those of the *WT* at 0 hours. The data were expressed as mean and SE. **P < 0.01 vs. the *WT* mice (*n* = 3–8 in each group). **D** RT-qPCR analysis of CD40 and Tissue factor in sorted liver sinusoidal endothelial cells. The gene expression levels were normalized to those of the *WT* at 0 hours. The data were presented as mean and SE (*n* = 3 in each group). *P < 0.05, **P < 0.01 vs. the *WT* mice. **E** The distribution of total nonparenchymal cells with c3 re-clustered is presented on the UMAP plot. The feature plots showed the expression of CD40 and CD40L. The heatmap presented the relative strength of the CD40-CD40L signaling network for each cluster. ALT, alanine aminotransferase; CD40L, CD40 ligand; Con A, concanavalin A; IFNγ, interferon-gamma; ILC, innate lymphoid cell; LDH, lactate dehydrogenase; NK cell, natural killer cell; NKT cell, natural killer T cell; PTAH, phosphotungstic acid-hematoxylin; RT-qPCR, quantitative reverse transcription polymerase chain reactions; Tgd cell, gamma delta T cell; TNFα, tumor necrosis factor-alpha; UMAP, uniform manifold approximation and projection; VEGF, vascular endothelial growth factor; WT, wild type

To evaluate the individual impact of TNFα and IFNγ on Con A-induced pathological changes, we utilized TNFα knockout (*Tnf*^–/–^) and IFNγ knockout (*Ifng*^–/–^) mice and compared them with wild type (*WT*) mice. The *Tnf*^–/–^ and *Ifng*^–/–^ mice demonstrated lower ALT levels than *WT* mice after Con A treatment (Fig. 3A). The hematoxylin and eosin (HE) and phosphotungstic acid-hematoxylin (PTAH) staining demonstrated a smaller necrotic area and reduced sinusoidal fibrin deposition in *Ifng*^–/–^ and *Tnf*^–/–^ mice compared to *WT* mice (Fig. 3B). While the TNFα expression was significantly decreased in *Ifng*^–/–^ mice, IFNγ expression was relatively increased in *Tnf*^–/–^ mice compared to *WT* mice (Fig. 3C). Notably, a marked increase of TF gene expression observed in *WT* and *Tnf*^–/–^ mice was completely inhibited in *Ifng*^–/–^ mice after Con A treatment. It is known that cell-cell interaction including binding via the CD40- CD40 ligand (CD40L) pathway is one of the critical initiation factors to induce TF gene expression in endothelial cells [21–23]. CD40 expression was certainly suppressed in *Ifng*^–/–^ mice compared to *WT* and *Tnf*^–/–^mice, and it shared a similar expression profile with TF. No significant difference between *Ifng*^–/–^, *Tnf*^–/–^, and *WT* mice were found in CD40L expression (Fig. 3C). The upregulation of cell adhesion molecules, including intracellular adhesion molecule 1 (ICAM1) and endothelial cell selectin (E-selectin) as well as chemokine (C-C motif) ligand 2 (CCL2) were significantly suppressed in *Tnf*^–/–^ (Fig. S5). The *Tnf*^–/–^ and *Ifng*^–/–^ mice showed poor induction of hypoxia-related genes such as LDH and VEGF (Fig. 3C). These findings indicated that IFNγ had a stronger impact on TF expression than TNFα in the Con A model. Since IFNγ signaling targeted vascular endothelial cells as described previously (Fig. 2D, E), we enriched liver sinusoidal endothelial cells (LSECs) from the livers of the Con A model using the anti-CD146 antibody [18] and evaluated CD40 and TF gene expressions. Compared to *WT* mice, the gene expressions of CD40 and TF were significantly inhibited in *Ifng*^–/–^ mice (Fig. 3D). NPCs at 1 hour and 3 hours after Con A treatment with C3 re-clustered were presented on the UMAP plot, and the CD40 signaling network was analyzed by ligand-receptor analysis (Fig. 3E). Cluster 3C (NKT cells) was identified to express CD40L and interacted with diverse cell types, which are supposed to express CD40, including cluster 4 (vascular endothelial cells), 10 and 11 (macrophages) (Fig. 3E). KEGG (Kyoto Encyclopedia of Genes and Genomes) pathway analysis of the gene clusters increased in cluster 4 (vascular endothelial cells of 3h) compared with cluster 1 (vascular endothelial cells of control and 1h) revealed that the upregulated genes in cluster 4 were significantly enriched in genes related to inflammation including NF-kappa B signaling pathway, which was the downstream target of CD40-CD40L interaction (Fig. S6).

### Induction of tissue factor by IFNγ and CD40 ligand in liver endothelial cells

**Fig. 4:**
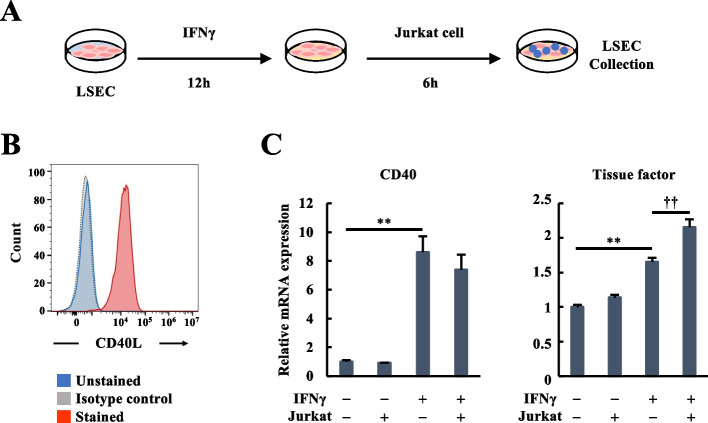
Induction of tissue factor by IFNγ and CD40 ligand in liver endothelial cells. **A** The LSEC were treated with 1,000 U/ml IFNγ for 12 hours and then incubated for 6 hours with Jurkat cells fixed with 1% paraformaldehyde. **B** Cell-surface expression of CD40L protein in untreated Jurkat cells by flow cytometry. The histograms depict isotype control (dashed line), unstained control (solid blue line), and CD40L expression (solid red line). The qualitatively identical results were obtained with at least three further batches of cells. **C** RT-qPCR analysis of CD40 and tissue factor in LSEC cells after IFNγ (1,000 U/ml) and Jurkat cell treatment. The gene expression levels were normalized to those of the IFNγ and Jurkat cell free group. The data were expressed as mean and SE (*n* = 5–10 in each group). **P < 0.01 vs. the IFNγ and Jurkat cell free group and ^††^*P* < 0.01 vs. the IFNγ group. CD40L, CD40 ligand; IFNγ, interferon-gamma; LSEC, liver sinusoidal endothelial cell; RT-qPCR, quantitative reverse transcription polymerase chain reactions

To confirm the IFNγ-CD40-TF axis in LSECs in vitro, the primary human LSECs were cultured with or without recombinant IFNγ and Jurkat cells fixed with paraformaldehyde (Fig. 4A). The constitutive expression of CD40L in unstimulated Jurkat cells was confirmed by flow cytometry (Fig. 4B). CD40 expression was notably elevated at 12 hours after IFNγ loading, regardless of the presence of Jurkat cells (Fig. 4C). In addition, TF was significantly upregulated by IFNγ stimulation, and further amplified by adding Jurkat cells (Fig. 4C). Even in the presence of TNFα, IFNγ with CD40L increased TF transcript (Fig. S7). These findings strongly suggest that IFNγ-mediated CD40 upregulation and the CD40-CD40L interaction enhance TF induction in LSECs.

### Significance of CD40 for microcirculatory disturbance in patients with acute liver injury

**Fig. 5:**
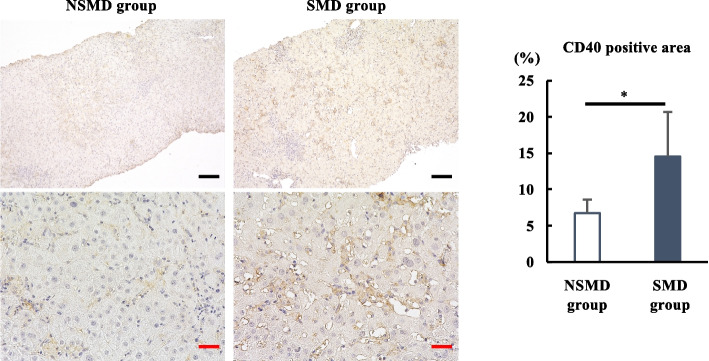
Significance of CD40 for microcirculatory disturbance in patients with acute liver injury. Liver sections from patients with acute liver injury were stained for CD40, and the positive areas were compared (*n* = 5 in each group). Black scale bars = 100 μm and red scale bars = 25 μm. *P < 0.05 vs. the SMD group. NSMD, non-sinusoidal microcirculatory disturbance; SMD, sinusoidal microcirculatory disturbance

Finally, to determine the significance of CD40 for the microcirculatory disturbance in clinical practice, CD40 expression was assessed by immunohistochemical analysis of liver biopsy samples from patients with ALI (Fig. 5). The majority of CD40-positive cells were LSECs, and the CD40-positive area was significantly more prominent in the SMD group with high serum IFNγ levels than in the NSMD group (Fig. 5), implying that the IFNγ-CD40 axis might be clinically crucial for the microcirculatory disturbance in ALI.

## Discussion

Here, we identified a precise mechanism of the IFNγ-CD40 axis by which immune cell activation triggered intrahepatic microcirculatory disturbance in ALI. IFNγ was secreted from ILCs stimulated by Con A and upregulated CD40 expression in LSECs. We demonstrated that CD40-CD40L interaction induced TF expression in LSECs, which led to thrombosis and microcirculatory disturbance.

IFNγ is known to play an essential role in tissue homeostasis as well as immune and inflammatory responses [24]. This cytokine is produced by immune cells, including innate-like lymphocyte populations such as NK cells and ILCs, as well as adaptive immune cells, such as T helper 1 (Th1) cells and CD8+ cytotoxic T lymphocytes (CTLs) [24]. IFNγ is associated with hepatic inflammation in patients with liver diseases. The hepatic infiltration of IFNγ positive lymphocytes was related to the inflammatory process in chronic hepatitis B patients [25], and IFNγ positive peripheral blood mononuclear cells (PBMCs) increased in acute exacerbation of hepatitis B [26]. In addition, the IFNγ expression in PBMCs was associated with inflammatory activity in chronic hepatitis C patients [27, 28], and serum IFNγ levels were elevated in fulminant hepatic failure patients [29]. However, the impact of IFNγ on the murine models of liver diseases is controversial. IFNγ promoted hepatic inflammation in the transgenic mice model of hepatitis B [30], acetaminophen-induced ALI [31], and Con A-induced ALI [18, 32]. In contrast, IFNγ had a protective effect on the CCl_4_ model [33] and cholestasis model by bile duct ligation [34]. These inconsistent results might be attributed to different cellular sources and targets of IFNγ depending on disease models. Therefore, we applied scRNA-seq and FACS analysis to the Con A-induced ALI model and identified that IFNγ secreted by ILCs activated LSECs (Fig. 2E). NKT cells were recognized as key effector cells during immune reactions and reported to be involved in IFNγ production and liver injury in the Con A-induced ALI [35–38]. The dose of Con A is known to affect the degree of liver injury after the injection [36], suggesting that the variation of dosage might provide different cellular environments and reactions. Moreover, it is reported that IFNγ secretion and liver injury in NKT cells deficient mice were suppressed at the later stage of Con A-induced ALI [38]. NKT cells were the major source of IFNγ in the unstimulated state and continuously expressed IFNγ, while the IFNγ upregulation was detected only in ILCs 1h after Con A treatment (Fig. 2C), indicating that ILCs, rather than NKT cells, were stimulated to produce IFNγ at the early stage of Con A-induced ALI. ILC has been recently proposed as a subset of immune cells with classical lymphoid cell architecture, which expresses no cell-surface markers that distinguish other immune cell types [39]. Nabekura et al. reported that ILCs were activated to secrete IFNγ in the CCl_4_-induced mouse ALI model, as our results [40]. However, they demonstrated that IFNγ exerted hepatoprotective effects by upregulating the Bcl-xl expression in hepatocytes. We considered that the different cellular targets of IFNγ (i.e., LSECs in the Con A model and hepatocytes in the CCl_4_ model) or the different degrees of liver injury might result in contradictory effects. On the other hand, TNFα is well known to be involved in ALI [41–44]. As expected, the serum TNFα level in patients with ALI was elevated compared to its normal level previously reported; however, it had no significant difference between the patients with sinusoidal microcirculatory disturbance (SMD group) and NSMD group (Fig. 1A). In addition, the hepatic gene expression of TNFα was significantly elevated in the TNF/GalN model, an ALI model without microcirculatory disturbance, compared to the control group; in contrast, IFNγ was not enhanced in the mice (Fig. 1B). Our scRNA-seq analysis revealed that the source of TNFα was resident macrophages at the early stages of liver injury and recruited macrophages at the later stages of liver injury (Fig. 2B). These findings indicated that TNFα secreted by macrophages has a more minor impact on microcirculatory disturbance in ALI than IFNγ secreted by ILCs.

Con A has previously been reported to bind to mannose-rich glycoproteins on resident macrophages and activate CD4^+^ T cells by crosslinking these cells via T-cell receptors [45, 46]. However, our scRNA-seq analysis revealed that ILCs and macrophages, rather than T cells, were initially activated and secreted IFNγ or TNFα respectively at 1 hour after Con A administration (Fig. 2A–F). The mechanisms by which Con A activates ILCs and macrophages are unknown and need to be elucidated. As the etiologies of ALI with microcirculatory disturbance are diverse, our finding that the initiators of the pathological changes in ALI were innate immune cells rather than adaptive immune cells seemed plausible.

It is known that endothelial cells express CD40, and CD40 expression is upregulated by IFNγ and TNFα in human umbilical vein endothelial cells (HUVECs) [22, 23, 47]. Furthermore, CD40L, which is generally considered to be expressed in CD4^+^ T cells, induces cell surface adhesion molecules and TF-dependent procoagulant activity of HUVECs via CD40 [22, 23, 47, 48]. However, whether the IFNγ-CD40-TF axis acts in LSECs in ALI with microcirculatory disturbance has been unclear. In this study, IFNγ depletion alleviated liver injury by inhibiting the upregulation of CD40, TF, and hypoxia-related genes, including LDH and VEGF, without affecting CD40L expression (Fig. 3A–C). In addition, TF was induced by IFNγ stimulation, and further amplified by CD40L interaction (Fig. 4C). These findings indicate that IFNγ plays a pivotal role in the microcirculatory disturbance in ALI by increasing the susceptibility of LSECs to CD40L. TNFα depletion demonstrated a minimal effect on CD40 and TF (Fig. 3C), which is contradictory to previous studies using HUVECs [22, 23]. The special characteristics of LSECs in cytokine reaction might provide these inconsistent findings. In contrast, the depletion of TNFα did alleviate Con A-induced liver injury concomitant with inhibition of the hypoxia-related gene expression (Fig. 3A–C). Considering the findings that TNFα secreted by macrophages targeted endothelial cells (Fig. 2E) and TNFα is known to directly affect endothelial barrier function to promote thrombogenesis [23], TNFα possibly contributed to sinusoidal hypercoagulation via other pathways than CD40-TF axis. Consistent with the report that TNFα induces sinusoidal hypercoagulation through upregulating the endothelial adhesion molecules [49], TNFα depletion reduced ICAM1 and E-selectin expression in this study (Fig. S5). Although other stimuli, including lipopolysaccharide, interleukin-1β, and VEGF, can elicit TF in endothelial cells, our in vitro study showed that IFNγ effectively induced CD40 and TF expression in LSECs regardless of TNFα presence, which implied IFNγ might be sufficient for developing sinusoidal hypercoagulation. Consequently, we assume that the IFNγ-CD40 axis is the link between immune cells and sinusoidal hypercoagulation and plays an essential role in the pathogenesis of ALI with microcirculatory disturbance.

This study confirmed that the serum IFNγ level and hepatic CD40 expression were significantly elevated in human ALI patients with sinusoidal microcirculatory disturbance (Figs. 1A and 5). Because a percutaneous liver biopsy for severe ALI patients is hazardous due to their marked coagulopathy, only a few studies reported histological analysis of severe human ALI. Nevertheless, the analogy between the Con A model and human ALI with microcirculatory disturbance indicated that immune cells and LSECs play a crucial role in human ALI. Because ALI with microcirculatory disturbance can occur regardless of etiologies, we speculate that the "etiologies" such as viruses, autoimmune reactions, and medications may trigger first-hit liver damage. And the innate immune responses to the first-hit liver damage may determine whether a self-limited ALI may proceed to severe ALI with microcirculatory disturbance. We assume that the dysregulation of ILCs and excessive IFNγ secretion are linked to the mechanisms. However, other cytokine pathways, other cell populations, or other backgrounds, including genetic susceptibility, epigenetic alternations, metabolic disorders, and the microbiome could also be involved in the process; these factors should be investigated in the future. Furthermore, we think that the classification of ALI patients based on the presence of microcirculatory disturbance is essential to examine the effect of medical treatment for ALI. Although several treatment procedures, such as anticoagulant therapy and steroids, were previously investigated for ALI [8, 50–53], the utility was not established. Given that anticoagulant therapy is inevitably effective for sinusoidal hypercoagulation, ALI patients should be classified according to microcirculatory disturbance to reassess the therapeutic effects of these treatments.

In this study, we revealed the critical roles of IFNγ and TNFα and their sources and downstream pathways. However, further investigation for other cytokines and cell-cell communications that contributed to developing microcirculatory disturbance in ALI should be conducted. In addition, this work focused on the early stages of Con A-induced ALI; however, it is usually difficult to administrate the treatment interventions for ALI patients in the early stage in clinical settings. Therefore, the mechanism of the sustainment of liver injury also needs to be studied.

## Conclusions

We revealed the pathogenesis of ALI with microcirculatory disturbance. This study may provide novel insights into basic and clinical research and potential therapies for ALF with microcirculatory disturbance.

## Methods

### Patients and samples

We conducted a single-center retrospective study on patients with ALI admitted at Kyushu University Hospital between April 2012 and March 2020. Patients with ALI who presented with serum ALT levels greater than 500 U/l were enrolled in the study, and serum samples were collected on admission (*n* = 120). Blood samples were used to assess the liver function, coagulation profile, immunological parameters, and viral markers such as HAV, HBV, HCV, HEV, cytomegalovirus, herpes simplex virus (types 1 and 2), and Epstein-Barr virus. At discharge, autoimmune hepatitis (AIH) diagnoses were confirmed according to the revised criteria of the International Autoimmune Hepatitis Group. Patients with malignant tumors and liver cirrhosis who had been previously diagnosed by blood tests or imaging were excluded from this study. Thirty patients received ultrasound-guided percutaneous liver biopsies. In this study, the normal ranges for ALT and LDH in humans were 6–30 U/l and 119–229 U/l, respectively, and the ALT/LDH ratio was calculated using the following formula: ALT/LDH ratio = (serum ALT – the upper limit of normal (ULN))/ (serum LDH – ULN). Using a serum ALT/LDH ratio of 1.5 as a cut-off value, the patients were classified into SMD group and NSMD groups, as previously described [10]. The background characteristics of patients with ALI are shown in Table 1. This study adhered to the Helsinki Declaration and was approved by the Kyushu University Hospital Ethics Committee (no. 27-377 and 2021-77). Due to the retrospective nature of this study, no informed consent was acquired from patients. The datasets used in this study are available in the repository of the Department of Medicine and Bioregulatory Science, Graduate School of Medical Sciences, Kyushu University.

### Animals and experimental protocols

Eight-week-old male C57BL/6J mice were obtained from Japan SLC (Shizuoka, Japan). The IFNγ knockout (*Ifng*^−/−^) mice and TNFα knockout (*Tnf*^−/−^) mice with C57BL/6 background were purchased from the Jackson Laboratory (Bar Harbor, ME). Mice were acclimated to the environment in a temperature, humidity, and light-controlled room (12 h light and 12 h dark cycle) and given free access to standard chow and water. Concanavalin A (Con A) was purchased from Sigma-Aldrich (St. Louis, MO) and dissolved in PBS. Con A (25 mg/kg) was injected intravenously into mice, and severe hepatitis without affecting mice mortality was induced. All animals were euthanized with isoflurane and sacrificed for sample collection 0, 1, 3, 6, and 12 hours after Con A administration. For TNFα/galactosamine (TNF/GalN)-induced ALI model, 700 mg/kg of GalN (Sigma-Aldrich, St. Louis, MO) was injected intraperitoneally, and one hour later, 15 μg/kg of TNFα (recombinant human TNFα; Peprotech, Cranbury, NJ) was injected intravenously through the tail vein. All animal studies were conducted in accordance with the Guide for the Care and Use of Laboratory Animals of the National Institutes of Health and were approved by the Animal Care Committee of Kyushu University.

### Cell culture

Human liver sinusoidal endothelial cells (LSECs) were purchased from LONZA (Walkersville, MD) and cultured on EGM™-2 BulletKit™ media at 37°C and 5% CO_2_. Jurkat cells, an immortalized line of human T lymphocyte cells (provided by RIKEN BRC), were cultured on Roswell Park Memorial Institute (RPMI) 1640 medium containing 10% fetal bovine serum (FBS), 100 U/ml penicillin and 100 µg/ml streptomycin. Recombinant human IFNγ and TNFα were obtained from PeproTech (Rocky Hill, NJ). LSEC were seeded in 12-well plates and cultured to confluence. Then, the cells were treated with cytokines (TNFα 1,000 U/ml and IFNγ 1,000 U/ml) for 12 hours and then incubated for 6 hours with Jurkat cells fixed with 1% paraformaldehyde. Then, the medium was removed, and the cells were washed twice and harvested for further analysis.

### Biochemical analysis

The mouse ALT levels were measured using the DRI-CHEM NS500sV kit (Fujifilm, Tokyo, Japan). The human serum TNFα and IFNγ levels were measured using the TNFα and IFNγ ELISA kits (catalog no. ab46087 and ab46025, respectively, Abcam, Cambridge, UK) according to the manufacturer’s protocols.

### Immunohistochemical analysis

The human liver biopsy samples were fixed with 10% formalin, embedded in paraffin, and cut into 5-μm serial sections. The paraffin-embedded tissue sections were deparaffinized, rehydrated, and pre-treated using heat-mediated antigen retrieval with EDTA (pH 9.0). Endogenous peroxidase activity was blocked for 30 minutes with 3% hydrogen peroxide (Sigma-Aldrich, St. Louis, MO). After 30 minutes of blocking with diluted serum from the secondary antibody host, the slides were incubated overnight at 4℃ with anti-CD40 antibody (catalog no. ab224639, Abcam, Cambridge, UK). Afterwards, the secondary goat anti-rabbit antibodies (catalog no. ab97051, Abcam, Cambridge, UK) were applied. The sections were incubated for 60 minutes at room temperature and then stained with diaminobenzidine tetrahydrochloride (Sigma-Aldrich, St. Louis, MO). The sections were counterstained with hematoxylin (Muto Pure Chemicals Co, Tokyo, Japan), dehydrated, and mounted. Then, the sections were examined under a Keyence BZ-X700 microscope (Keyence, Osaka, Japan). The antibody-positive areas in five randomly-selected microscopic fields (magnification ×100) per section were measured using analysis software (BZ-X analyzer, Keyence, Osaka, Japan), and the mean percentage of the antibody-positive area was calculated. The mouse liver tissue samples were fixed in 10% formalin and embedded in paraffin. The general histological characteristics were identified using hematoxylin and eosin (HE) staining, while the sinusoidal fibrin deposition was detected using phosphotungstic acid-hematoxylin (PTAH) staining.

### Quantitative reverse transcription polymerase chain reaction

The total RNA was extracted from liver tissue or LSECs using TRIzol reagent (Invitrogen, Carlsbad, CA), and cDNA was synthesized using the PrimeScript RT Master Mix kit (Takara Bio, Tokyo, Japan). The quantitative reverse transcription polymerase chain reactions (RT-qPCRs) were performed using the TB Green Premix Ex Taq II kit (Takara Bio, Tokyo, Japan). The target gene expression was normalized to glyceraldehyde 3-phosphate dehydrogenase (GAPDH) expression, and relative expression was calculated using the 2^−ΔCt^ method. The primer sequences used in this study are listed in Table S1.

### Isolation of hepatic nonparenchymal cells

Following the exposed portal vein cannulation, the mouse liver was pre-perfused in situ with Hanks' balanced salt solution containing 0.5 mM of EGTA and 0.01 M of 4-(2-hydroxyethyl)-1-piperazineethanesulfonic acid to remove blood from the liver. Next, the liver was perfused with collagenase solution (Dulbecco's Modified Eagle Medium supplemented with 2% FBS, 0.1 mg/ml collagenase I+II) and then mechanically disrupted. The cell suspension was placed in a collagenase solution with 1,000 U/ml DNase I and incubated for 30 minutes at 37℃ with shaking for digestion. To remove hepatocytes, cells were filtered through a cell strainer with 100-μm pore nylon mesh (FALCON, Corning, NY) and centrifuged at 90 × g for 3 minutes. Then, the supernatant was centrifuged at 570 × g for 5 minutes, and the pellet containing hepatic nonparenchymal cells (NPCs) was resuspended in ACK (ammonium-chloride-potassium) lysing buffer (Gibco, NY, USA) for 3 minutes to lyse red blood cells. After washing with PBS to stop the lysis, the cells were resuspended with 25% Percoll (Cytiva, Uppsala, Sweden) in RPMI 1640 medium, layered on a 65% Percoll solution, and then centrifuged for 15 minutes at 570 × g at room temperature. The interphase between both Percoll layers containing NPCs was gently transferred into a new tube, diluted with FACS buffer (PBS supplemented with 2% of FBS, 2 mM EDTA, and 0.1% NaN3), and centrifuged at 400 × g for 3 minutes at 4 ℃ to pellet the cells. The trypan blue staining was performed to assess the cell viability.

### Flow cytometry

The freshly isolated NPCs were resuspended in FACS buffer at a 1 million cells/ml concentration. The cells were preincubated with an anti-CD16/32 monoclonal antibody to avoid the nonspecific antibody binding to the Fc receptor. For detection of surface markers, cells were stained with anti-CD45 BV510, anti-CD3 FITC, anti-CD4 APC/Cy7, anti-CD8 BV785, anti-NK1.1 APC, anti-NK1.1 PE/Cy5, anti-CD11b BV421, anti-F4/80 APC/Cy7, anti–CD146 FITC, or isotype-matched control immunoglobulin (all were supplied from BioLegend, San Diego, CA). For intracellular IFNγ-mRNA staining, PrimeFlow RNA™ assay (Thermo Scientific, Waltham, MA) was used according to the manufacturer's protocol. The stained cells were analyzed and sorted using a BD FACSMelody cell sorter (BDbiosciences, Franklin Lakes, NJ), and the data were processed using the Flow Jo software (Tree Star Inc., Ashland, OR).

### Single-cell RNA sequencing analysis

The single-cell RNA sequencing analysis (scRNA-seq) was performed according to the previously described protocol [54]. A sequence-ready library was generated using the Illumina TruSeq™ library prep kit (Illumina, San Diego, CA) following the manufacturer’s instructions. The scRNA-seq libraries were sequenced using an Illumina NovaSeq 6000 S4 reagent kit (300 cycles) (Illumina, San Diego, CA) in paired-end sequencing mode (read1:75 cycles, read2: 225 cycles). The barcode sequences were extracted from the read 1 fastq files. The read 2 fastq files, which included each cell’s mRNA, were directly aligned to Refseq transcript sequences using bowtie 2.2.6. The aligned reads were matched to their paired extracted barcode sequences. The gene count data in individual cells were collected by counting mapped reads per barcode. All samples were analyzed with the R package Seurat 4.0.4 [55]. The cell doublets and low-quality cells were filtered using a unique molecular count threshold greater than 2,000 or less than 200. Low-quality cells with more than 9% mitochondrial counts were filtered out. Then all samples were normalized and scaled with the Seurat NormalizeData (normalization.method = "LogNormalize", scale.factor = 10000) and ScaleData (features = all.genes) functions, respectively. All cells from all three datasets were first clustered using the Seurat FindNeighbors (dims = 1:14) and FindClusters (resolution = 0.3) functions. All clusters were labeled with cell types using the R packages SingleR 1.6.1 and celldex 1.2.0 [56] with a reference dataset, the ImmGen database (available at http://www.immgen.org/). Highly expressed genes were visualized with the Seurat FeaturePlot function. The R package CellChat 1.1.3 was used to enable a systematic analysis of cell-cell communication and to explore the ligand-receptor pairs between cell groups at different times [57]. Functional enrichment analyses were performed using the database for annotation, visualization, and integrated discovery (DAVID) (v6.8) [58, 59].

### Statistical analysis

The data were analyzed using the JMP Pro, version 16 statistical software (SAS Institute Inc., Cary, NC). The results were expressed as means and standard deviation (SD) or standard errors of the mean (SEM). The unpaired Student’s t-test was used to determine whether significant differences were found between groups, while the one-way ANOVA and Tukey’s post hoc tests were used to compare the means among multiple groups. A *p*-value of less than 0.05 indicated statistical significance.

### Supplementary Information

**Supplementary Material 1.**

**Supplementary Material 2.**

## Data Availability

The dataset used and/or analyzed during the current study is available from the corresponding author upon reasonable request.

## Abbreviations

ALF: Acute liver failure
ALI: Acute liver injury
ALT: Alanine aminotransferase
CD40L: CD40 ligand
Con A: Concanavalin A
GalN: Galactosamine
IFNγ: Interferon-gamma
ILC: Innate lymphoid cell
LDH: Lactate dehydrogenase
LSEC: Liver sinusoidal endothelial cell
NK cell: Natural killer cell
NKT cell: Natural killer T cell
NPC: Nonparenchymal cell
NSMD: Non-sinusoidal microcirculatory disturbance
RT-qPCR: Quantitative reverse transcription polymerase chain reaction
scRNA-seq: Single-cell RNA sequencing analysis
SMD: Sinusoidal microcirculatory disturbance
TF: Tissue factor
TNFα: Tumor necrosis factor-alpha
WT: Wild type
