# Supplementary figures and images for: Microcirculatory disturbance in acute liver injury is triggered by IFNγ-CD40 axis
Source: J Inflamm (Lond). 2024 Jun 21;21:23. doi: 10.1186/s12950-024-00387-w (PMC11191181; doi:10.1186/s12950-024-00387-w)

**Fig. S1**

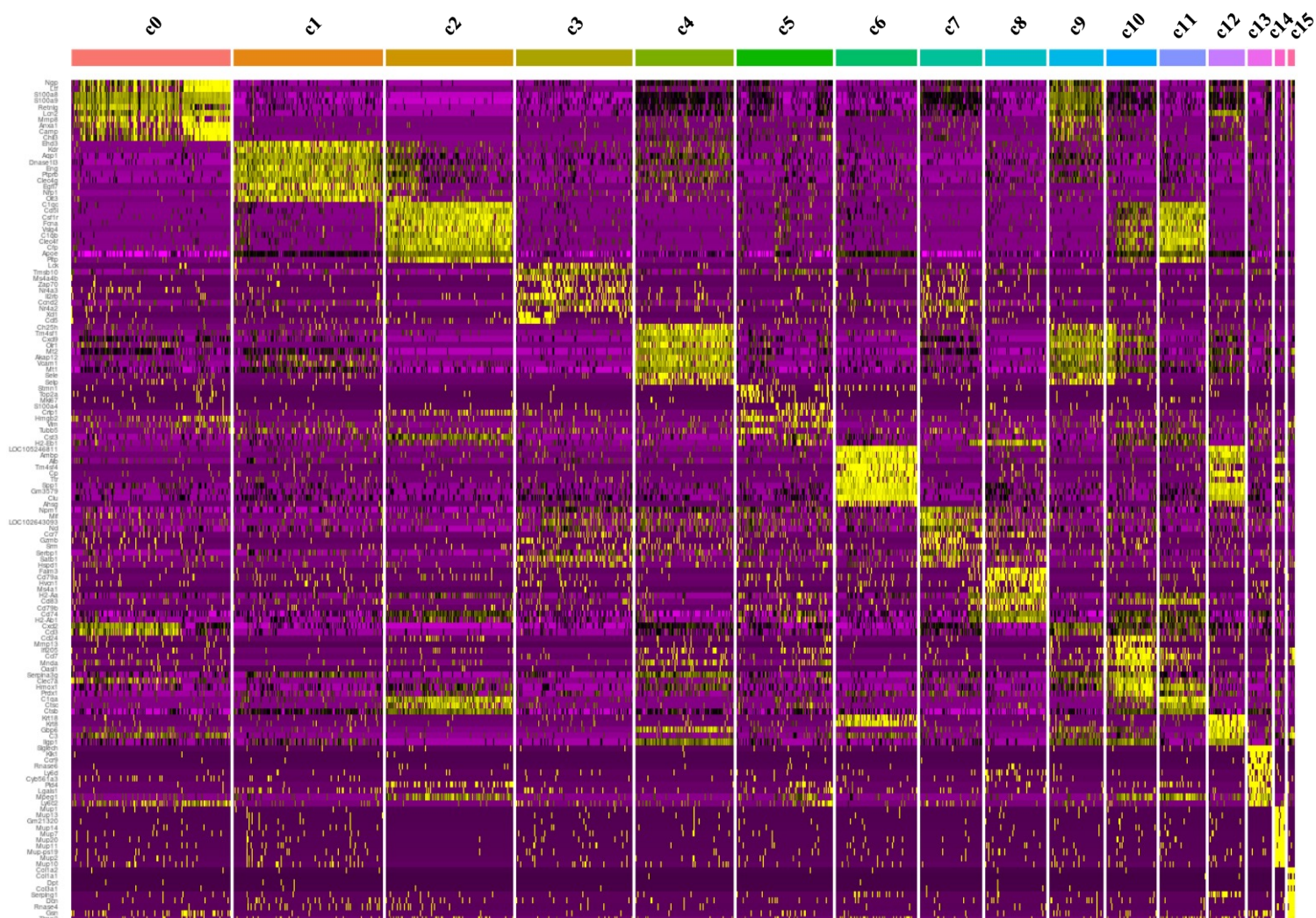

Fig. S2

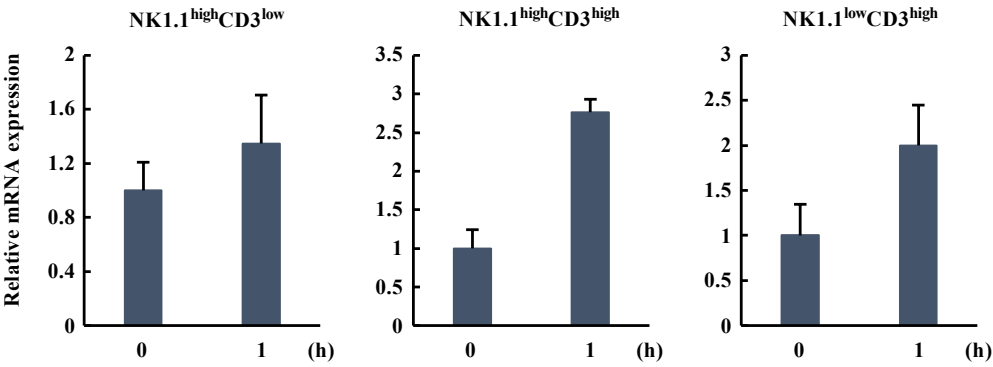

Fig. S3

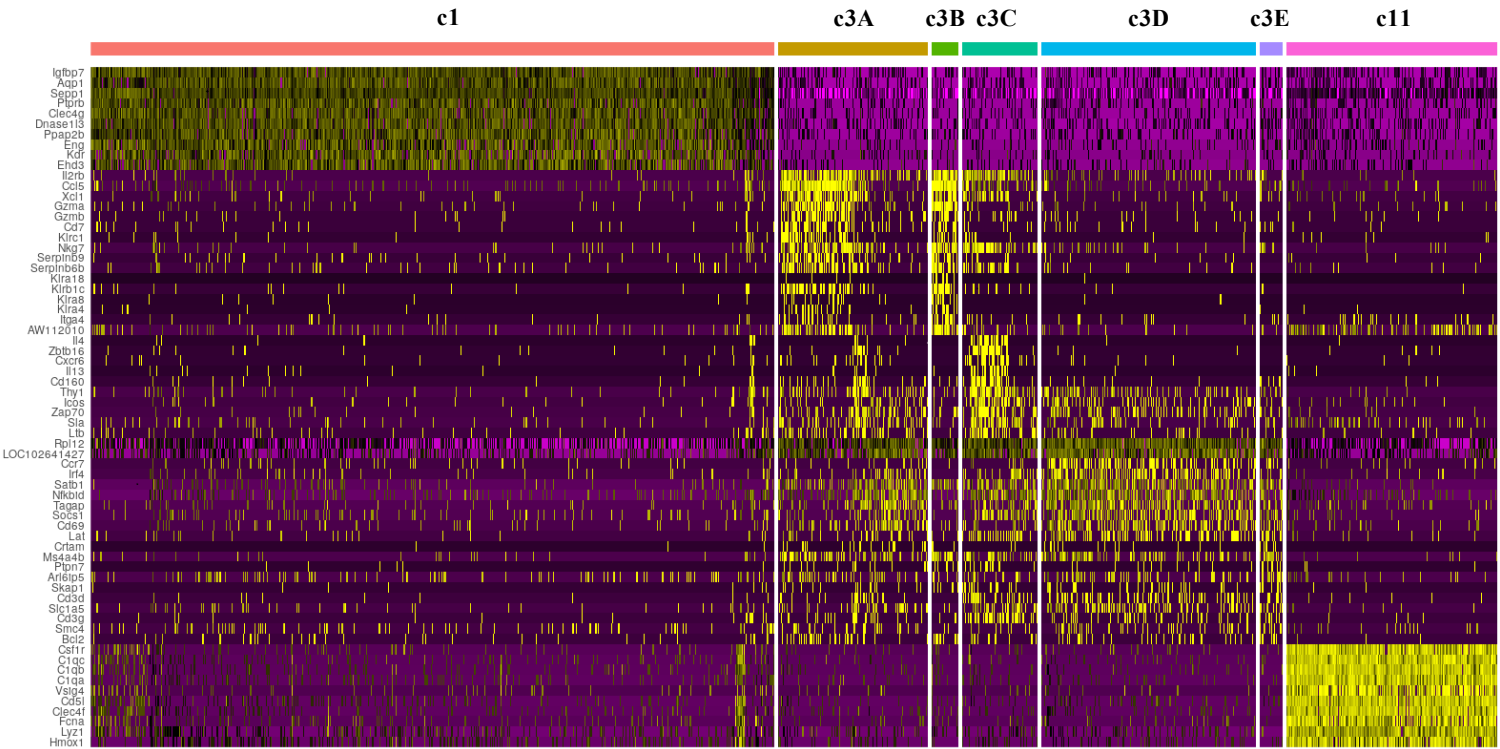

Fig. S4

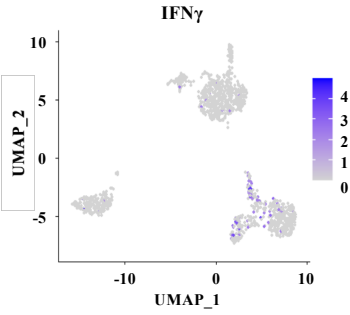

Fig. S5

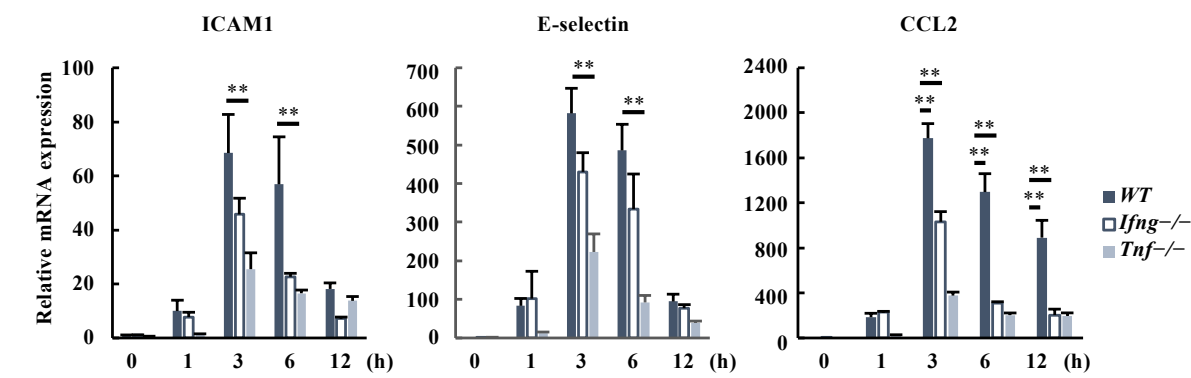

Fig. S6

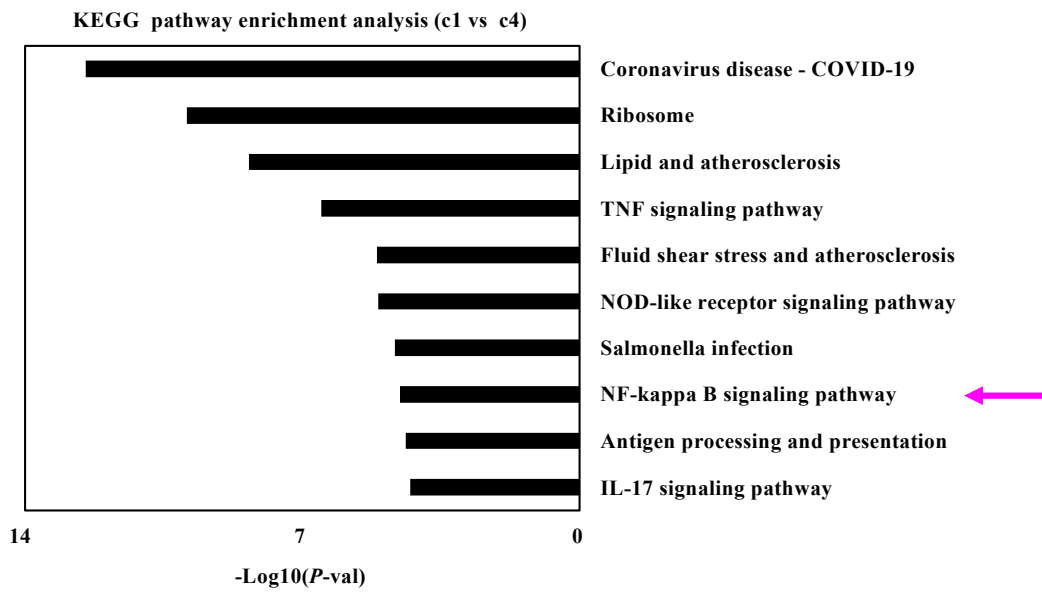

Fig. S7

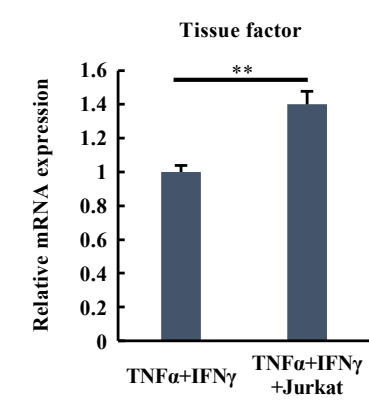

Supplement: Supplementary file 1 — Supplementary Material 1. [file 12950_2024_387_MOESM1_ESM.pdf]
