# Supplementary material for: Microcirculatory disturbance in acute liver injury is triggered by IFNγ-CD40 axis
Source: J Inflamm (Lond). 2024 Jun 21;21:23. doi: 10.1186/s12950-024-00387-w (PMC11191181; doi:10.1186/s12950-024-00387-w)
Supplement: Supplementary file 2 — Supplementary Material 2. [file 12950_2024_387_MOESM2_ESM.docx]

**Table S1. Primers’ sequences used in the present study.**

| Genes | Gene name | Forward (5'–3') | Reverse (5'–3') |
| --- | --- | --- | --- |
|  |  | Human primers |  |
| GAPDH | *GAPDH* | ATCACCATCTTCCAGGAGCGA | TTCTCCATGGTGGTGAAGACG |
| CD40 | *CD40* | CCTGTTTGCCATCCTCTTGGTG | AGCAGTGTTGGAGCCAGGAAGA |
| TF | *F3* | ATTACAGGTGCCGCACTACC | GATTGAAGTCAGCCCTCAA |
|  |  | Mouse primers |  |
| GAPDH | *Gapdh* | TGTGTCCGTCGTGGATCTGA | TTGCTGTTGAAGTCGCAGGAG |
| TNFα | *Tnf* | AGGCACTCCCCCAAAAGATG | TGAGGGTCTGGGCCATAGAA |
| IFNγ | *Ifng* | CTGCCACGGCACAGTCATTG | TCTGGCTCTGCAGGATTTTCA |
| TF | *F3* | TGCTTCTCGACCACAGACAC | TAAAAACTTTGGGGCGTTTG |
| LDH | *Ldha* | TGGCAGCCTCTTCCTTAAAA | TTTTCCAAGCCACGTAGGTC |
| VEGF | *Vegfa* | ACACGGTGGTGGAAGAAGAG | GGAAGGGAAGATGAGGAAGG |
| HO-1 | *Hmox1* | ACGCATATACCCGCTACCTG | AAGGCGGTCTTAGCCTCTTC |
| CD40 | *CD40* | GCTATGGGGCTGCTTGTTGA | ATGGGTGGCATTGGGTCTTC |
| CD40L | *CD40lg* | GTGAGGAGATGAGAAGGCAA | CACTGTAGAACGGATGCTGC |
| ICAM1 | *Icam1* | GGGCTGGCATTGTTCTCTAA | CTTCAGAGGCAGGAAACAGG |
| E-selectin | *Sele* | ACGCAAGTTCTCCAGCTGTT | AGCTACCCATGGAACACGAC |
| CCL2 | *Ccl2* | AGGTCCCTGTCATGCTTCTG | GCTGCTGGTGATCCTCTTGT |

CCL2, chemokine (C-C motif) ligand 2; CD40L, CD40 ligand; E-selectin, selectin, endothelial cell; GAPDH, glyceraldehyde-3-phosphate dehydrogenase; HO-1, heme oxygenase 1; ICAM1, intercellular adhesion molecule 1; IFNγ, interferon-gamma; LDH, lactate dehydrogenase; TF, Tissue factor; TNFα, tumor necrosis factor-alpha; VEGF, vascular endothelial growth factor.
